# Erythropoietin Produces a Dual Effect on Carotid Body Chemoreception in Male Rats

**DOI:** 10.3389/fphar.2021.727326

**Published:** 2021-09-14

**Authors:** Christian Arias-Reyes, Sofien Laouafa, Natalia Zubieta-DeUrioste, Vincent Joseph, Aida Bairam, Edith M. Schneider Gasser, Jorge Soliz

**Affiliations:** ^1^ Université Laval, Faculté de Médecine, Centre de Recherche de l’Institut Universitaire de Cardiologie et de Pneumologie de Québec, Département de Pédiatrie, Québec, QC, Canada; ^2^ High Altitude Pulmonary and Pathology Institute (HAPPI-IPPA), La Paz, Bolivia; ^3^ Institute of Veterinary Physiology, Vetsuisse-Faculty, University of Zurich, Zurich, Switzerland; ^4^ Center for Neuroscience Zurich (ZNZ), Zurich, Switzerland

**Keywords:** chemosensing, hypoxia, hypercapnia, nitric oxide, electrophysiology

## Abstract

Erythropoietin (EPO) regulates respiration under conditions of normoxia and hypoxia through interaction with the respiratory centers of the brainstem. Here we investigate the dose-dependent impact of EPO in the CB response to hypoxia and hypercapnia. We show, in isolated “*en bloc*” carotid body (CB) preparations containing the carotid sinus nerve (CSN) from adult male Sprague Dawley rats, that EPO acts as a stimulator of CSN activity in response to hypoxia at concentrations below 0.5 IU/ml. Under hypercapnic conditions, EPO did not influence the CSN response. EPO concentrations above 0.5 IU/ml decreased the response of the CSN to both hypoxia and hypercapnia, reaching complete inhibition at 2 IU/ml. The inhibitory action of high-dose EPO on the CSN activity might result from an increase in nitric oxide (NO) production. Accordingly, CB preparations were incubated with 2 IU/ml EPO and the unspecific NO synthase inhibitor (L-NAME), or the neuronal-specific NO synthase inhibitor (7NI). Both NO inhibitors fully restored the CSN activity in response to hypoxia and hypercapnia in presence of EPO. Our results show that EPO activates the CB response to hypoxia when its concentration does not exceed the threshold at which NO inhibitors masks EPO’s action.

## Introduction

The carotid body (CB) is the main chemo-sensor located at the bifurcation of the carotid arteries (Ortega-Saenz and Lopez-Barneo, 2020). It primarily detects small arterial changes of partial pressure of oxygen (PO_2_)_,_ and secondarily, detects changes in carbon dioxide (PCO_2_) and pH levels. In turn, the CB activates the respiratory center in the brainstem to elicit proper adaptive ventilatory responses (Peng et al., 2010; Kumar and Prabhakar, 2012).

The CB is composed of clusters of glomus cells (the chemoreceptive elements of carotid bodies) in close contact with the blood vessels and the sinus nerve. Glomus sensory (type 1) cells contain O_2_ and CO_2_ sensitive K+ channels which are inhibited by hypoxia or hypercapnia (Lopez-Barneo et al., 2001; Weir et al., 2005). The inhibition of K^+^ channels leads to cell depolarization, Ca^2+^ entry (Buckler and Vaughan-Jones, 1994; Urena et al., 1994), and the release of neurotransmitters (Leonard et al., 2018), including biogenic amines (dopamine, catecholamines); acetylcholine; neuropeptides; and adenosine triphosphate (ATP) (Prabhakar and Overholt, 2000; Bairam and Carroll, 2005). Such increased neurotransmitter release stimulates the carotid sinus nerve (CSN) activity that leads to increased ventilation in response to hypoxia and hypercapnia (Ding et al., 2011; Del Rio et al., 2017; Ortega-Saenz and Lopez-Barneo, 2020). Completing the machinery, the CB glomus cells also synthesize nitric oxide (NO), which is not stored in vesicles but functions as a chemical messenger that inhibits the CB excitation induced by hypoxia and consequently the ventilatory response (Prabhakar, 1994). Both nitric oxide synthase, endothelial (eNOS) and neuronal (nNOS-3) were proposed as possible sources of NO in CB (Gozal et al., 1996; Dvorakova and Kummer, 2005; Li et al., 2010; Atanasova et al., 2016).

The hypoxia-inducible hormone erythropoietin (EPO) and EPO receptor (EPOR) have been found in catecholaminergic glomus cells type I and PC12 cells (Sasaki and Burrows, 1998; Soliz et al., 2005). EPO signaling in PC12 cells, and likely in glomus cells type I, leads to membrane depolarization, activation of Ca^2+^ channels, increase of tyrosine hydroxylase activity, dopamine biosynthesis, and NO production (Koshimura et al., 1999), and consequently regulates the CSN activity. Indeed, systemic EPO stimulates carotid body chemosensory activity following hypoxic and hypercapnic stimulation (Soliz et al., 2005; Gassmann et al., 2010; Andrade et al., 2018b). Complementary, it has been shown that both chronic intermittent hypoxia (1 min cycle between air and 5% O_2_, 8 h/day for 3–28 days) and chronic sustained hypoxia (10% O_2_, for 3–28 days) upregulate the expression of EPO and its receptor in the carotid body of rats (Lam et al., 2009).

Although increasing evidence suggests that EPO is a positive modulator of hypoxic and hypercapnic chemosensitivity of the carotid body, divergent results have recently been reported (Andrade et al., 2018a; Andrade et al., 2018b). Systemic injection of high EPO doses (2,000 IU/Kg) to anesthetized Sprague Dawley rats exposed to hypoxia failed to stimulate CSN discharges in females and males and did not affect the chemosensory response to hypercapnia in males (Andrade et al., 2018a; Andrade et al., 2018b). Therefore, these results suggest a dose-response effect of EPO on the CB activation upon hypoxia and hypercapnia.

In this study, we investigated the EPO-mediated dose-dependent response of CBs exposed to hypoxia and hypercapnia. To do so, we determined a threshold for EPO stimulatory response to hypoxia and hypercapnia in *ex vivo* “*en bloc*” carotid body preparations from Sprague Dawley male adult rats. Recordings of CSN activity were obtained after 60 min incubation of the CB at different concentrations of recombinant human EPO (0.1, 0.2, 0.5, 1, and 2 IU/ml). Additionally, we assessed whether the inhibitory effect of high EPO dose in the CSN activity results from inhibition by NO derived from carotid body type I cells. Our results show that EPO stimulates the hypoxic response to hypoxia in males at low concentrations (<0.5 IU/ml) and inhibits CSN activity at hypoxia and hypercapnia at high concentrations (>1 IU/ml) due to an increase in NO production from type I cells. EPO’s induced CSN activity shows a PO_2_ afferent response within a physiological range of arterial PO_2_. Thus, the application of very high doses of EPO in clinics suggests caution, especially when patients have low O_2_ saturation.

## Materials and Methods

### Animals

Female and male Sprague-Dawley rats used for mating were obtained from Charles River Canada (St-Constant, QC, Canada). The experiments were conducted on rats that were born and raised in our animal care facilities. The animals received food and water ad libitum and were kept under standard laboratory and animal care conditions (21°C, 12:12 dark/light cycle; lights on at 07:00, and off at 19:00). *Ex vivo* preparations of mono lateral CB from adult male rats (3–4 months of age) were used to perform electrophysiological recordings. A total of 5–6 animals and carotid body bifurcations per group were used. Animal experiments were performed following the ARRIVE guidelines and approved by the Laval University Animal Care Committee from Université Laval (VRR-18-073).

### Carotid Bifurcation Extraction and Perfusion

**FIGURE 1:**
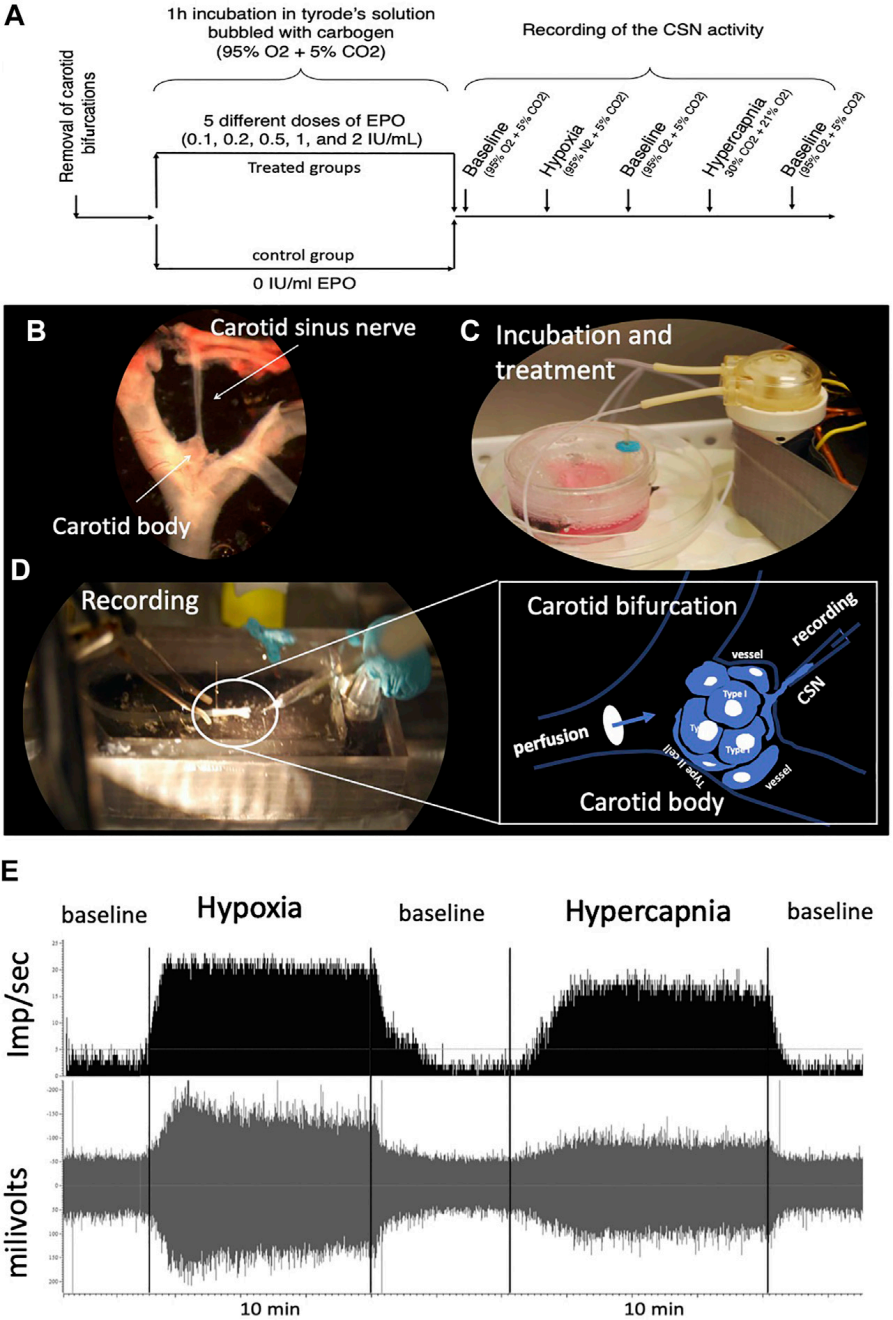
**(A)** Schematic representation of the experimental design. One EPO concentration per experimental group was used. **(B)** “*en bloc*” dissection of the carotid bifurcation containing the carotid body and the carotid sinus nerve (CSN). **(C)** An electric peristaltic minipump was used to perfuse the carotid bifurcation into a tyrode solution during incubation of the sample with (or without, control) EPO. **(D)** The carotid bifurcation was catheterized during the recording of the CSN activity; The framed diagram is a schematic representation of the perfusion system of the CB by catheterization of the carotid bifurcation and the recording of the activity of the CSN through a suction electrode. **(E)** typical recording of CSN activity under baseline, hypoxic and hypercapnic conditions.

Male rats were deeply anesthetized with ketamine (80 mg/kg, i.p.)/xylazine (10 ml/kg, i.p.), both carotid bifurcations were removed “*en bloc*” (Figures 1A,B). The CSN was then carefully dissected and cleared of the surrounding connective tissue. The dissected preparation was placed in a Petri dish containing 5 ml of ice-cold Tyrode’s solution (in mM: 125 NaCl, 5 KCl, 2 MgSO_4_, 1.2 NaH_2_PO_4_, 25 NaHCO_3_, 1.8 CaCl_2_, 5 sucrose and 10 glucose, pH 7.4) bubbled with carbogen (95% O_2_ + 5% CO_2_), where was incubated for 60 min at different doses of EPO (recombinant human EPO; CliagAG, Canada), 0 IU/ml (control group); 0.1 IU/ml; 0.2 IU/ml; 0.5 IU/ml; 1 IU/ml; and 2 IU/ml (Figures 1A,C). The perfusion system was kept at 36°C in a water bath. To enhance the contact of the EPO with the cells of the carotid body during the incubation time, a perfusion circuit was established using an electric peristaltic pump and a catheter with one end in the perfusion solution and the other catheterizing the common carotid artery. Perfusion was kept at a constant level of 6 ml/min (Figure 1C).

### *Ex Vivo* Recording of the CSN Activity

Standard methods were used to record action potentials from the carotid sinus nerve *ex vivo* (Iturri et al., 2015; Soliz et al., 2016). Briefly, the preparation was deposited in the recording chamber (volume = 5.4 ml) and a catheter was placed between the infusion solution inlet and the common carotid artery (Figures 1A,D). Care was taken to keep the recording chamber at 36°C (TC2Bip temperature controller; Cell Micro-Controls; Norfolk, VA, United States). The infusion rate was kept constant at 6 ml/min. The tip of a glass electrode (Model No. 573000, A-M Systems Inc., Carlsborg, WA) was used to suck up the CSN to record its activity. The tip of the electrode was sealed by applying sufficient suction to seal the electrode against the connective tissue surrounding the junction of the carotid body and the CSN. A grounding electrode was inserted into the recording chamber. The current electrical signal was connected to a differential input stage preamplifier. It was filtered and amplified (200 gain; x10 amplification; 30–1,500 Hz filtration) (Neurolog modules NL100AK, NL104A, NL126, NL106). The signal was processed by an A/D converter (Micro 1401-2 Cambridge Electronic Design (CED), Cambridge, United Kingdom) to display raw activity and frequency histograms on a computer running the Spike 2 software (CED). Chemoreceptor discharges were discriminated in Spike 2 as action potentials with an amplitude 25% above the reference noise and that responded to a decrease in PO_2_ and an increase in PCO_2_ of perfusion with a reversible increase in frequency.

### Experimental Protocol

The effect of EPO on CSN activity was evaluated as follows: the experiment started when a stable CSN discharge rate was observed under the initial conditions for at least 10 min (Tyrode’s solution bubbled with 95% of O_2_ + 5% CO_2_). The baseline recording was then held for an additional 5 min before switching to a Tyrode solution previously equilibrated with a hypoxic gas mixture (95% N_2_ + 5% CO_2_) that was held for 8–10 min. The preparation was then returned to basal conditions (95% O_2_ + 5% CO_2_) for about 5–7 min. When activity returned to baseline, the recording was started under hypercapnic stimulation by perfusion with Tyrode’s solution bubbled with 30% CO_2_, 21% O_2_, N_2_ balanced. This stimulus was maintained for 8–10 min before returning to initial conditions (Figure 1A). These perfusion protocols were obtained from previous similar works (Pepper et al., 1995; Cummings and Wilson, 2005; Peng and Prabhakar, 2018). Each carotid body preparation was used for a complete protocol. Figure 1E shows a typical control recording of CSN activity under baseline, hypoxic and hypercapnic conditions.

### Data Analysis and Statistics

The CSN activity was assessed by measuring the number of impulses above threshold per seconds on a second-by-second basis. Initially, baseline activity was calculated for the baseline and recovery periods by averaging values over 150 s. The plateau of the response was obtained by averaging the activity over a 500–700 s period under hypoxic and hypercapnic conditions according to our previous protocol (Iturri et al., 2015).

All statistical analyses were performed using GraphPad Prism version 9.1 for Windows, GraphPad Software, San Diego, California United States, www.graphpad.com. Prior conducting 2-way ANOVA tests on the baseline and plateau’s data we explored normality and homogeneity of variance of the residuals via Q-Q and homoscedasticity plots. No substantial diversions were observed. EPO’s impact on maximal CSN activity during hypoxia and hypercapnia was analyzed for each independent EPO dose using 2-way ANOVA.

For curve-fitting analyses on ramp and recovery data we tested normality of the residuals via Shapiro-Wilk tests and Q-Q plots. Homoscedasticity was verified using the analysis included in PRISM and homoscedasticity plots. We observed no relevant deviations from assumptions. For the recovery data during hypercapnic exposure, the distribution of the residuals was not normal, however, this is a condition that can be ruled out in this type of analysis, particularly when the differences in slopes are very clear. The effect of stimuli on the response during the ramp and recovery phases was evaluated by extra sum-of-squares F tests, comparing the corresponding Hill slopes obtained from nonlinear regressions. The data was fit using a four-parameters sigmoidal model:

Y=Bottom+(Top-Bottom)*(t^Hillslope)/((Et50^Hillslope) + (t^Hillslope)) 

where:

Bottom: mean value of the bottom section of the curve.

Top: mean value of the top section of the curve.

t. time.

Hill slope: slope factor that indicates the steepness of the curve.

Et50: the time when Y has reached the half point between Bottom and Top.

The method of fitting was least squares regression and a maximum of 1,000 iterations were performed.

All results were reported as mean ± SD. *p* < 0.05 was considered statistically significant.

## Results

### Inverted U-Shape EPO Dose-Response Curve of the Carotid Sinus Nerve Activity Under Hypoxia

**FIGURE 2:**
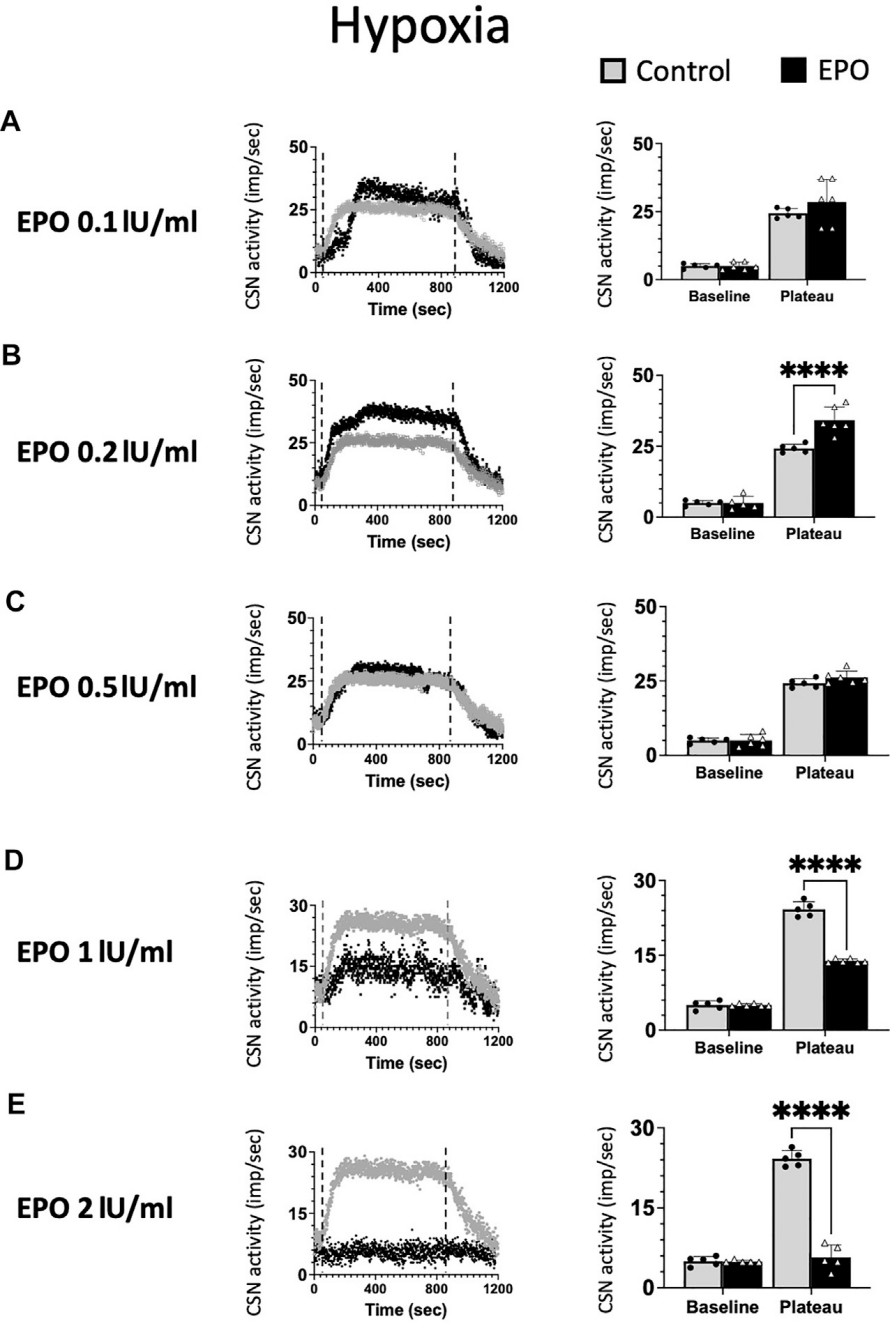
Erythropoietin (EPO) induces dose-dependent carotid sinus nerve (CSN) activity under hypoxic conditions. EPO at a concentration of 0.2 IU/ml significantly increases the activity of CSN (RM Two-way ANOVA F (1, 8) = 28.6 *p* = 0.0007) **(B)**, while there was no difference between the control and EPO at a concentration of 0.1 IU/ml (RM Two-way ANOVA F (1, 9) = 1.47 *p* = 0.256) **(A)** nor 0.5 U/ml (RM Two-way ANOVA F (1, 9) = 2.04 *p* = 0.187) **(C)**. Erythropoietin (EPO) at a concentration of 1 IU/ml leads to a strong inhibition of CSN activity (RM Two-way ANOVA F (1, 9) = 189 *p* < 0.0001) **(D)** and 2 IU/ml supress the activity of the carotid sinus nerve (CSN - RM Two-way ANOVA F (1, 8) = 199 *p* < 0.0001) **(E)**. Dashed lines indicate the beginning and end of the hypoxic stimulus. ****: *p* < 0.0001.

We tested *ex vivo* the impact of five EPO concentrations: 0.1; 0.2; 0.5; 1; and 2 IU/ml in the response of the CSN to hypoxia (Figure 2). Changes in maximum CSN activity (imp/sec) between control and EPO treated samples were analyzed by 2-way ANOVA. Compared to the control group, a significant increase in the CSN activity upon hypoxic stimulus was observed with EPO concentration at 0.2 IU/ml (RM Two-way ANOVA F (1, 8) = 28.6 *p* = 0.0007, Figure 2B). with no significant changes at 0.1 IU/ml (RM Two-way ANOVA F (1, 9) = 1.47 *p* = 0.256, Figure 2A) and 0.5 IU/ml (RM Two-way ANOVA F (1, 9) = 2.04 *p* = 0.187, Figure 2C).

At 1 IU/ml concentration, the response to hypoxia was reduced compared to control (RM Two-way ANOVA F (1, 9) = 189 *p* < 0.0001, Figure 2D). Moreover, 2 IU/ml of recombinant human EPO completely blunted the carotid body response to hypoxia (Figure 2E, RM Two-way ANOVA F (1, 8) = 199 *p* < 0.0001).

Our results show a threshold in the excitatory response of chemosensory cells to varying doses of EPO. High EPO concentrations reverse the effect of EPO from excitatory to inhibitory under hypoxia.

### High EPO Dose (>1 IU/ml) Inhibits the Carotid Sinus Nerve Activity in Response to Hypercapnia

**FIGURE 3:**
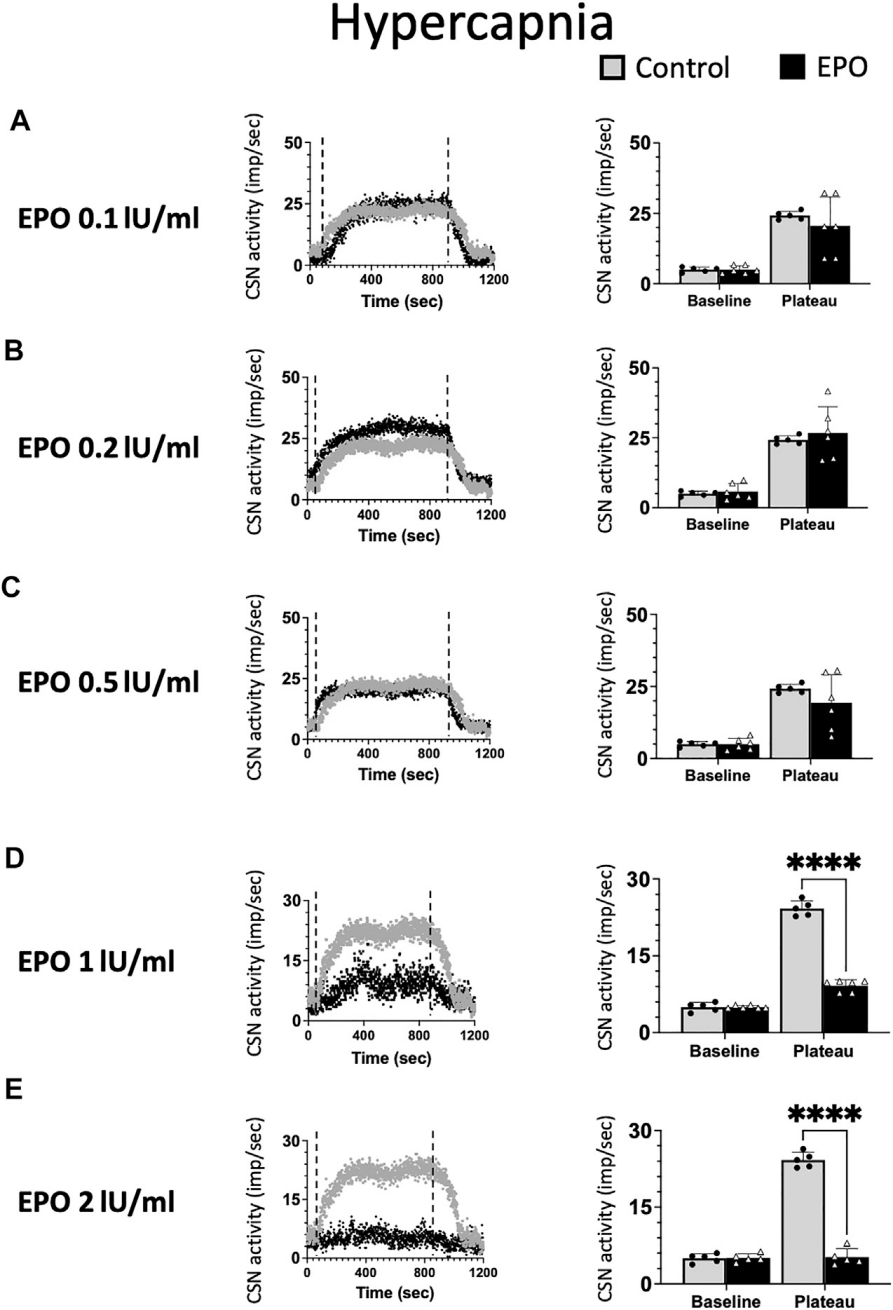
No significant impact of Erythropoietin (EPO) was evidenced at concentrations of 0.1 **(A)**, 0.2 **(B)** and 0.5 **(C)** IU/ml under hypercapnic conditions (RM Two-way ANOVA F_0.1_ (1, 9) = 0.744 *p* = 0.411; F_0.2_ (1, 9) = 0.194 *p* = 0.670; and F_0.5_ (1, 9) = 0.985 *p* = 0.347). At a concentration of 1 IU/m. EPO leads to a strong inhibition of CSN activity (RM Two-way ANOVA F (1, 9) = 257 *p* < 0.0001) **(D)** and 2 IU/ml supress the activity of the carotid sinus nerve (CSN - RM Two-way ANOVA F (1, 8) = 311 *p* < 0.0001) **(E)**. Dashed lines indicate the beginning and end of the hypercapnic stimulus. ****: *p* < 0.0001.

As for hypoxia, we tested the impact of five EPO concentrations (0.1; 0.2; 0.5; 1; and 2 IU/ml) on the response of the CSN to hypercapnia (Figure 3). EPO had no discernible effect at concentrations below 1 IU/ml (Figures 3A–C, RM Two-way ANOVA F_0.1_ (1, 9) = 0.744 *p* = 0.411; F_0.2_ (1, 9) = 0.194 *p* = 0.670; and F_0.5_ (1, 9) = 0.985 *p* = 0.347). At high EPO concentrations (1, and 2 IU/ml) EPO inhibited the CSN response to hypercapnia (Figure 3D, RM Two-way ANOVA F_1_ (1, 9) = 257 *p* < 0.0001 and Figure 3E, RM Two-way ANOVA F_2_ (1, 8) = 311 *p* < 0.0001).

In line with previously reported data (Ballot et al., 2015), our results show EPO to stimulate CSN activity only under hypoxia but not under hypercapnia. Additionally, high EPO concentrations are inhibitory.

### EPO Alters the CSN Activation Time to Changes in Oxygen and Carbon Dioxide

**FIGURE 4:**
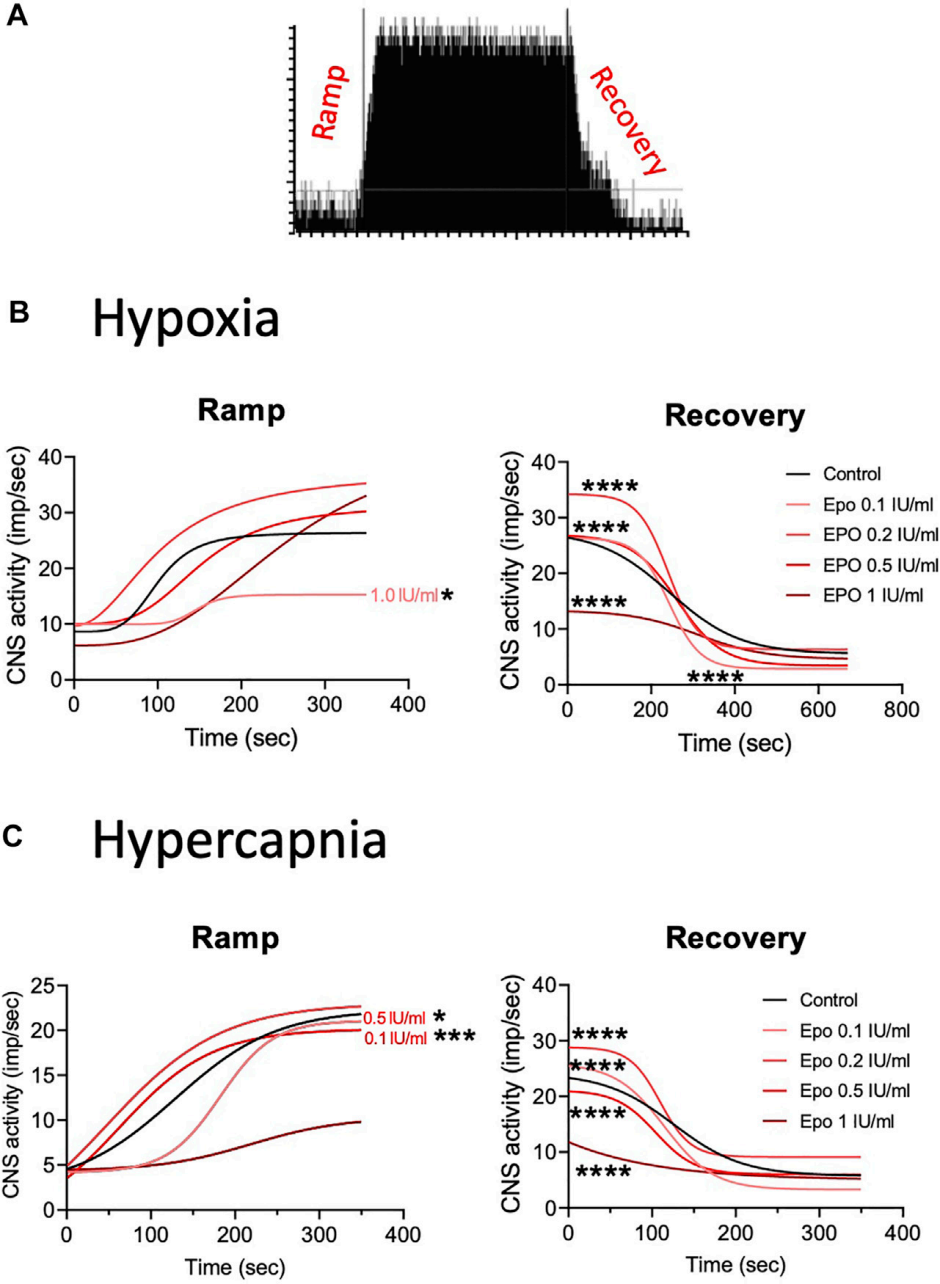
**(A)** Representation of activation (ramp) and deactivation (recovery) of the CSN due to changes in oxygen and carbon dioxide. **(B)** Regression curves applied to the stimulation (Ramp) and de-stimulation (Recovery) phases in the CSN response to hypoxia. In the stimulating phase, 1.0 IU/ml of EPO decrease the reactivity of the CSN while all the other concentrations of EPO produce no change in comparison to controls. During de-stimulation, all EPO concentrations significantly affect the slope in comparison to the control. **(C)** Regression curves applied to the stimulation (Ramp) and de-stimulation (Recovery) phases in the CSN response to hypercapnia. Significant differences in slope occur in Ramp for EPO concentrations of 0.1, and 0.5 1 IU/ml. In Recovery, all EPO concentrations produced significant changes in slope compared to controls.

We calculated the Hill slope of the sigmoid-fit curve to evaluate the time required to achieve maximal CSN stimulation (Ramp) with hypoxia or hypercapnia and the time to return to baseline (Recovery) (Figure 4A). Compared to control, EPO at concentrations of 0.1, 0.2, and 0.5 IU/ml did not lead to any significant change in the Ramp slope. While EPO at a concentration of 1 IU/ml significantly decreased the Ramp slope to hypoxia (Figure 4B left panel; Table 1A, F_1.0(1, 2451)_ = 3.928, *p* = 0.048). Under the recovery phase, compared to control, the slope was steeper with 0.1, 0.2, and 0.5 IU/ml of EPO, but flatter with 1 IU/ml (Figure 4B right panel; Table 1A, F_0.1 (1, 6187)_ = 199.7, *p* < 0.0001; F_0.2 (1, 6382)_ = 174, *p* < 0.0001; F_0.5 (1, 7921)_ = 765.6, *p* < 0.0001; F_1.0 (1, 5854)_ = 449.5, *p* < 0.0001). This result indicates a faster deactivation of the CSN after hypoxia when preparations were incubated with EPO at doses below 1 IU/ml.

**TABLE 1:** Effect of stimuli on the response of the carotid body during the ramp and recovery phases evaluated by comparing the Hill slopes for each curves. Significant *p* values (in bold) correspond to asterisques in Figure 4.

A) Hypoxia						
Ramp						
					Dif. vs. Control (Hill slope)	
EPO (IU/ml)	Bottom	Top	Hill slope	R^2^	F (df1, df2)	*p*
Control	8.65	26.42	4.53	0.97		
0.1	6.15	44.08	2.92	0.95	1.681 (1,2931)	0.195
0.2	9.7	37.34	2.08	0.94	1.01 (1,1702)	0.315
0.5	10.03	31.27	3.53	0.96	1.94 (1,2913)	0.16
1	9.93	15.28	9.57	0.56	3.928 (1,2451)	**0.048**

Recovery						
					Dif. vs. Control (Hill slope)	
EPO (IU/ml)	Bottom	Top	Hill slope	R^2^	F (df1, df2)	*p*
Control	4.25	25.6	−3.17	0.97		
0.1	2.5	26.1	−6.35	0.97	199.7 (1,6187)	**<0.0001**
0.2	6.01	33.8	−6.61	0.98	174 (1,6382)	**<0.0001**
0.5	2.92	26.1	−5.08	0.98	765.6 (1,7921)	**<0.0001**
1	4.17	12.9	−4.03	0.64	449.5 (1,5854)	**<0.0001**

B) Hypercapnia						
Ramp						
					Dif. vs. Control (Hill slope)	
EPO (IU/ml)	Bottom	Top	Hill slope	R^2^	F (df1, df2)	*p*
Control	5.24	24.3	2.48	0.94		
0.1	3.39	22.8	4.74	0.93	12.23 (1,3428)	**0.0005**
0.2	9.46	29.3	1.74	0.91	1.46 (1,3341)	0.23
0.5	5.53	20.8	1.25	0.9	1.25 (1,2808)	**0.02**
1	4.62	11.3	3.64	0.55	1.06 (1,3628)	0.81

Recovery						
					Dif. vs. Control (Hill slope)	
EPO (IU/ml)	Bottom	Top	Hill Slope	R^2^	F (df1, df2)	*p*
Control	4.57	22.8	−3.13	0.939		
0.1	2.68	24.9	−4	0.901	704.4 (1,4661)	**<0.0001**
0.2	8.87	28.5	−5.93	0.962	273.8 (1,4186)	**<0.0001**
0.5	5.67	20.5	−4.51	0.943	592.4 (1,4953)	**<0.0001**
1	3.42	11.8	−1.13	0.483	461.8 (1,4471)	**<0.0001**

In response to hypercapnia, compared to the respective control, EPO at concentrations of 0.1 and 0.5 IU/ml produced a significantly steeper slope, while EPO concentrations of 0.2, and 1 IU/ml showed no differences (Figure 4C left panel; Table 1B, F_0.1 (1, 3428)_ = 12.23, *p* = 0.0005; F_0.5 (1, 2808)_ = 5.25, *p* = 0.02). During the recovery phase, compared to control, EPO at concentrations of 0.1, 0.2 and 0.5 IU/ml increased the slope, while Epo at 1 IU/ml decreased it (Figure 4C right panel; Table 1B, F_0.1 (1, 4661)_ = 704.4, *p* < 0.0001; F_0.2 (1, 4186)_ = 273.8, *p* < 0.0001; F_0.5 (1, 4953)_ = 592.4, *p* < 0.0001; F_1.0 (1, 4471)_ = 461.8, *p* < 0.0001). These results suggests that EPO below 1 IU/ml increases the sensitivity of the CB to hypoxia and hypercapnia allowing a faster response of the CSN.

### High-Dose EPO-Mediated Inhibition of CSN Activity in Hypoxic and Hypercapnic Conditions is Prevented With NOS Inhibitors

**FIGURE 5:**
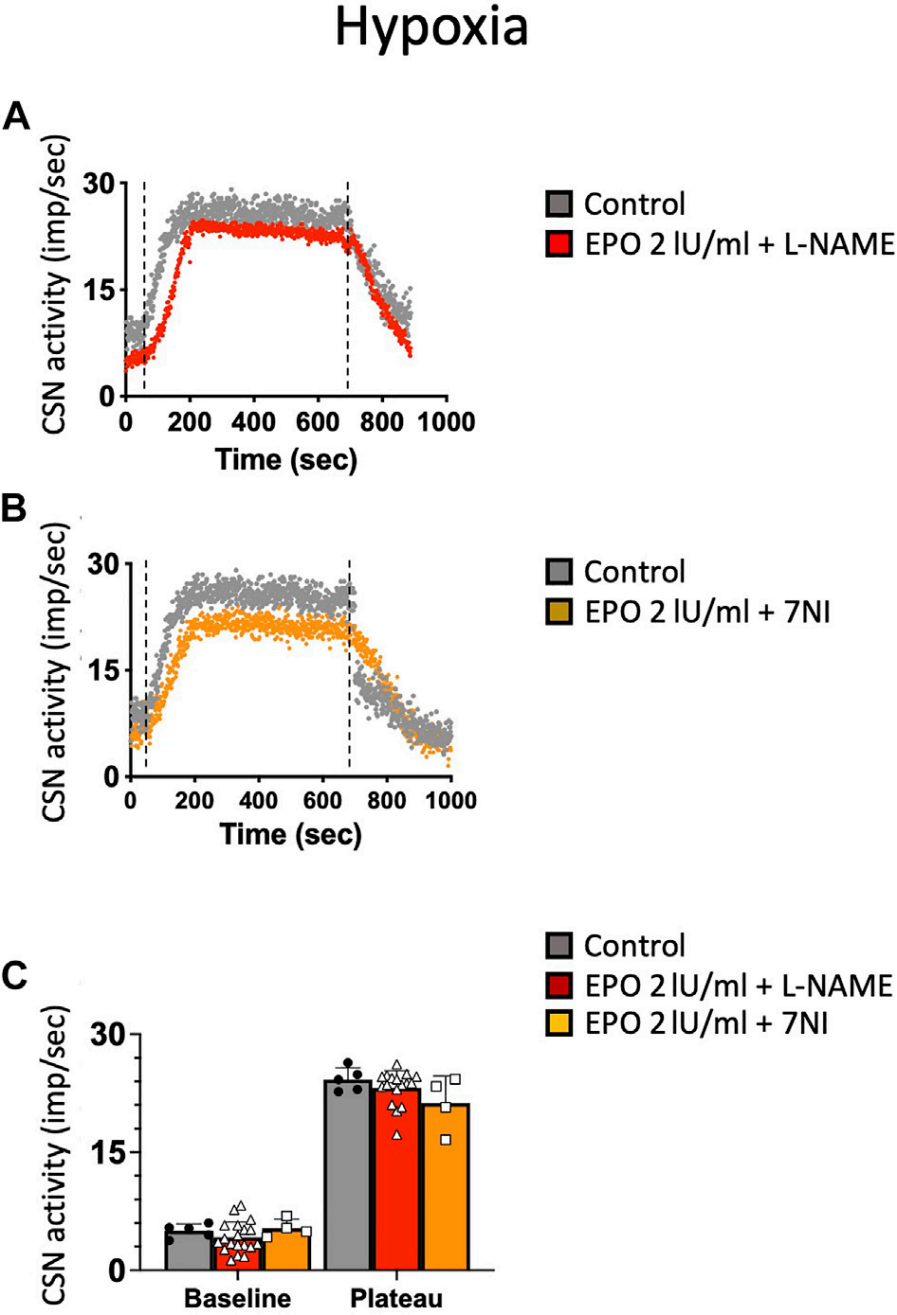
**(A)** Non-specific (L-NAME) and **(B)** neuronal-specific (7Ni) inhibitors of NOS prevented EPO-mediated inhibition of carotid sinus nerve (CSN) hypoxic activity at a concentration of 2 IU/ml **(C)** RM Two-way ANOVA F (2, 23) = 2.11 *p* = 0.144. Dashed lines indicate the beginning and end of the hypoxic stimulus.

**FIGURE 6:**
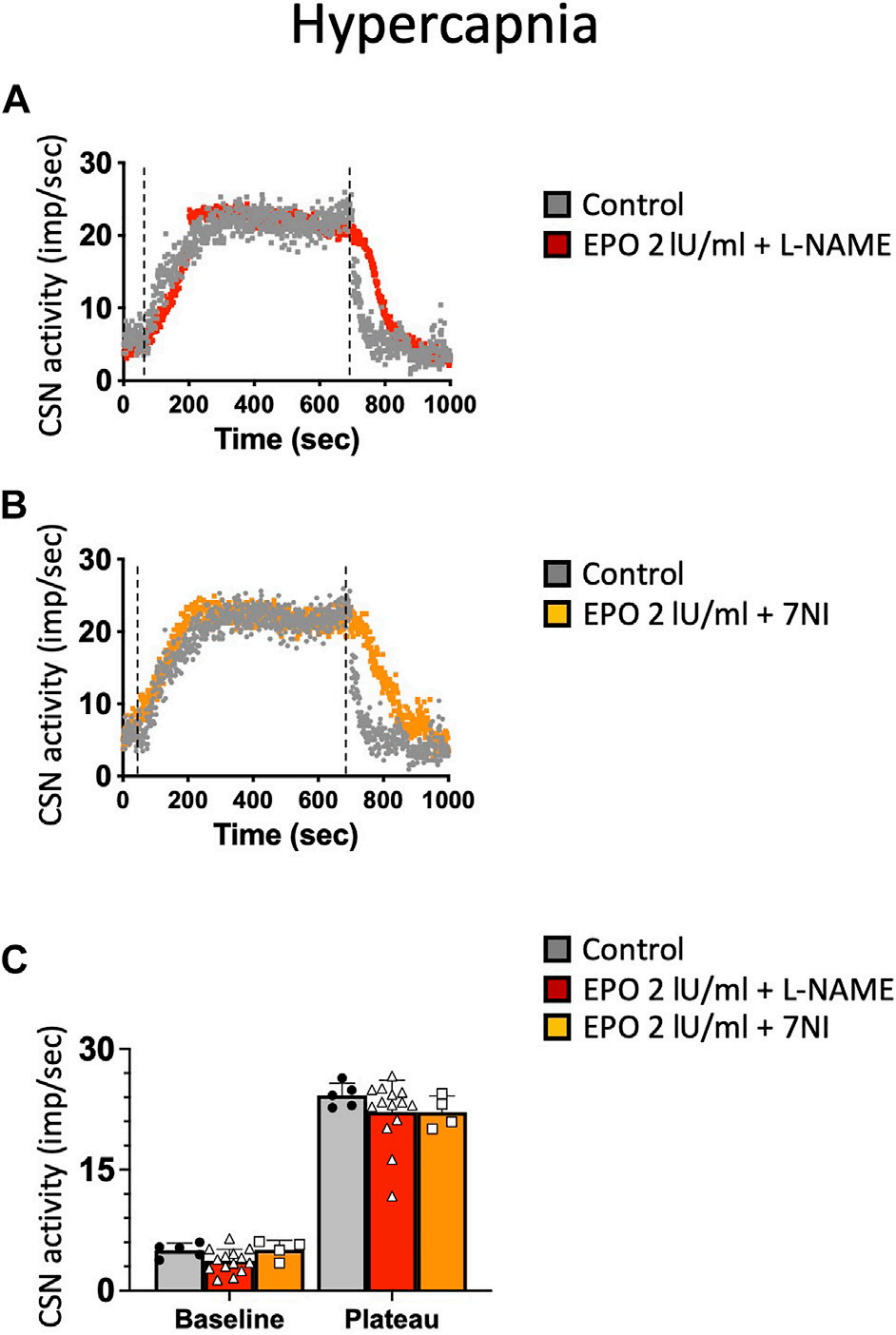
**(A)** non-specific (L-NAME) and **(B)** neuronal-specific (7Ni) inhibitors of NOS prevented EPO-mediated inhibition of carotid sinus nerve (CSN) hypercapnic activity at a concentration of 2 IU/ml.**(C)** RM Two-way ANOVA F (2, 20) = 0.424 *p* = 0.66. Dashed lines indicate the beginning and end of the hypoxic stimulus.

NO is an inhibitory messenger in the carotid body (Wang et al., 1995; Fung et al., 2001; Moya et al., 2012). EPO stimulates NO production in PC12 cells (Koshimura et al., 1999) and vascular endothelial cells (Beleslin-Cokic et al., 2004). Therefore, we hypothesized that the hypoxic and hypercapnic inhibition of CSN activity caused by high concentrations of EPO resulted from an increase in NO production from the carotid body type I cells and surrounding endothelial cells acting on CB chemosensitivity. To test this hypothesis, recordings with EPO (2 IU/ml) and NO inhibitors L-NAME (a non-specific inhibitor of NOS; 2 mM), or 7-nitroindazole (7NI - a specific inhibitor of neuronal, NOS; 2 mM) were done. Our results showed that both L-NAME and 7NI prevented the EPO-mediated inhibition of the CSN activity under hypoxia (RM Two-way ANOVA F (2, 23) = 2.11 *p* = 0.144, Figure 5) and hypercapnia (RM Two-way ANOVA F (2, 20) = 0.424 *p* = 0.66, Figure 6). These results confirm the hypothesis that 2 IU/ml of EPO promotes NO production in the carotid body cells type I, which in turn blunt the CSN response to hypoxia and hypercapnia.

## Discussion

**FIGURE 7:**
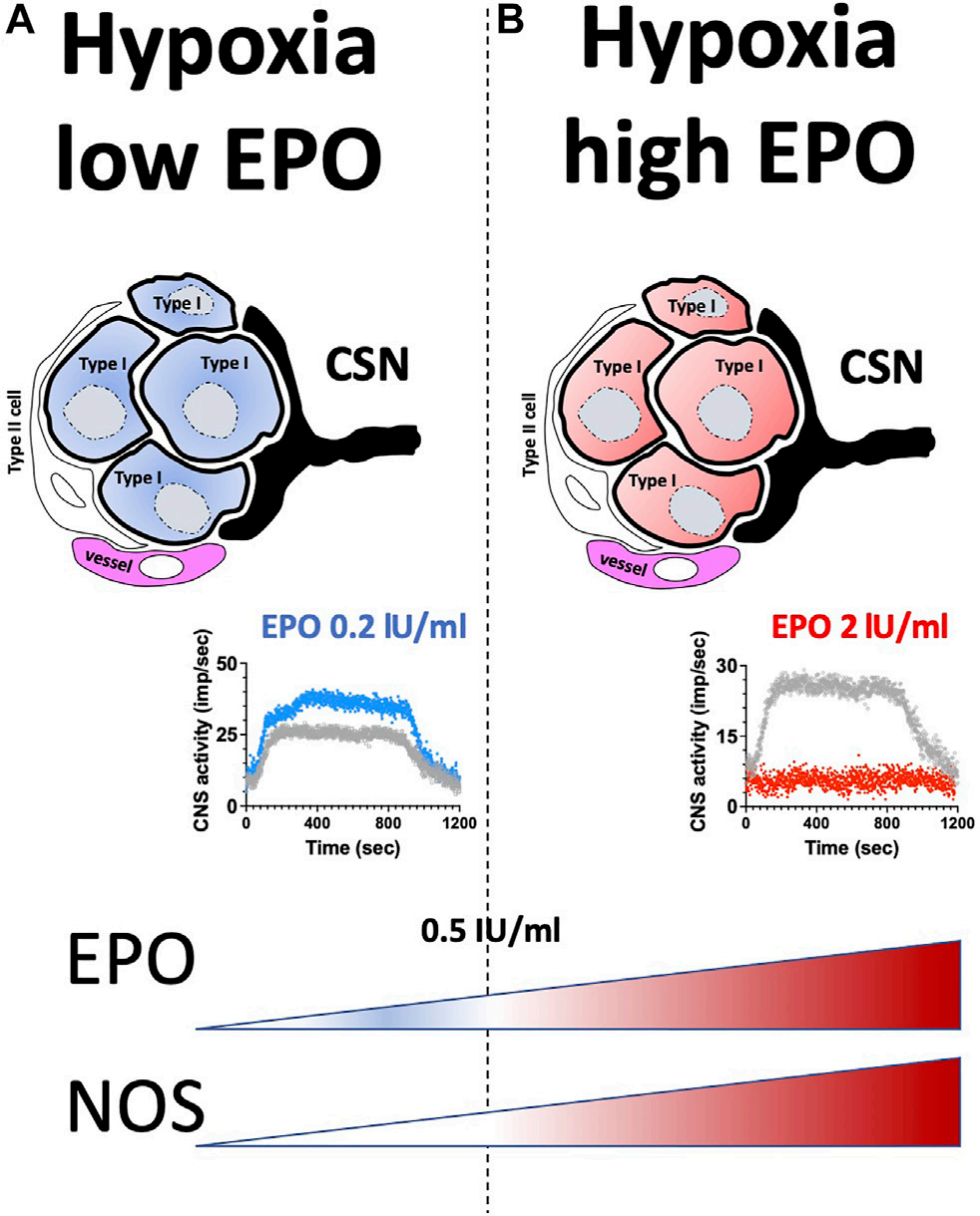
Graphical abstract. **(A)** low EPO concentration; **(B)** high EPO concentration.

Results of this study show a dual effect of EPO in the CB response to hypoxia and hypercapnia. Low EPO concentrations of 0.2 IU/ml increased the CSN activity (about 40%) under hypoxic, but not hypercapnic stimuli. EPO concentrations higher than 0.5 IU/ml progressively decreased hypoxic and hypercapnic CSN activation, causing complete inhibition with 2 IU/ml EPO concentration. NOS blockers prevented the EPO (2 IU/ml) mediated inhibition of hypoxic and hypercapnic CSN activity. Our results suggest that EPO stimulates sensory activity in response to hypoxia within a specific concentration range (0.1–0.5 IU/ml). When EPO concentration exceeds 1 IU/ml the suppression of the sensory responses to hypoxia and hypercapnia, apparently due to an exacerbated production of NO (Figure 7).

We previously showed that EPO modulates the neural control of normoxic and hypoxic ventilation by directly interacting with the respiratory centers of the brainstem (Soliz et al., 2005; Soliz et al., 2007; Khemiri et al., 2011; Ballot et al., 2015; Caravagna et al., 2015). Apart from central regulation of ventilation, a central question also was to determine whether EPO interacts with carotid bodies and its consequence on the activity of the CSN. Our data strongly support the hypothesis that EPO modulates the chemosensing function of carotid bodies. This is further supported by the wide distribution of EPORs in the islets of carotid body chemosensitive cells (Soliz et al., 2005; Gassmann et al., 2009); the altered ventilatory response to severe hypoxia (6% O_2_) in mice receiving intravenous injection of EPO (2,000 IU/kg) (Soliz et al., 2005; Soliz et al., 2007); and the upregulation of EPO and EPOR expression in the carotid body of rats after intermittent and chronic hypoxia (Lam et al., 2009). Furthermore, new data from recent reports also suggested that EPO modulates the chemosensory response of CB. Studies in rats showed that EPO increases the maximum CB response to hypercapnia in females (but not males), despite that no effects of EPO on the response to hypoxia were detected (Andrade et al., 2018b). Moreover, treatment of rats exposed to intermittent chronic hypoxia with carbamylated EPO promoted beneficial cardiorespiratory effects, mediated, at least in part, by attenuation of the CB chemoreresponse to hypoxia (Andrade et al., 2018a). Together, these studies suggested that EPO could be used as a target drug to protect against the harmful consequences of sleep-disordered breathing (Andrade et al., 2021). Our results show that there is an effect of EPO in modulating the response of the carotid body to stimulation under conditions of hypoxia but not to hypercapnia in male rats. Furthermore, our data reveal that the effect of EPO on the response of the hypoxic carotid body is dose-dependent. Concentrations between 0.1 and 0.5 IU/ml of EPO stimulate CSN activity, however, concentrations of EPO greater than 0.5 IU/ml inhibit the CSN hypoxic response. Besides, a parallel analysis shows that EPO alters the response time of CB to oxygen and CO_2_ changes (ramping, recovery), likely suggesting that EPO modulates the affinity of the O_2_/CO_2_ sensors (whatever their nature), thus modulating in turn cell depolarization and neurotransmitter release. We additionally showed that EPO does not stimulate CSN response to hypercapnia, and at concentrations higher than 1 IU/ml inhibits the response. In a previous study, we showed that intra-cisternal injection of EPO does not stimulate central chemosensitivity in response to hypercapnia (Ballot et al., 2015), direct EPO microinjections in the locus coeruleus (a central CO_2_/pH chemoreceptor site in mammals) attenuates the hypercapnia-induced hyperpnea (Silva et al., 2017). Thus, we suggest here that the inhibitory effect of EPO in response to CO_2_ is also related to the chemosensing machinery, blocked by high NO production.

The differences found in the effects of EPO on hypoxia and hypercapnia between different research groups may relay in the experimental design. Experiments carried out in anesthetized rats with the barbituric sodium pentobarbital (40 mg/kg i.p), may cause depression in the neural ventilatory regulation, which could mask the effects of the EPO both *in vivo* and *in vitro*. Secondly, different EPO concentrations and EPO sources may influence the half-life time of the hormone and consequently its effect on the CB and CSN. Finally, the percentage of O_2_ and CO_2_ used to induce hypoxia and hypercapnia varies across experiments. In our current experiments, we have a closed circuit in which perfusion of EPO goes directly to the carotid body and CSN, being an advantage to evaluate the direct effect of the hormone on the system. Nevertheless, our study cannot be compared to *in vivo* studies in which the physiology is preserved (Andrade et al., 2018a; Andrade et al., 2018b).

We show that EPO generates inhibition of the response of the carotid body to hypoxic and hypercapnic stimuli by increasing the production of NO, since inhibition of NO with L-NAME (non-specific inhibitor of NOS) and 7NI (a specific inhibitor of neural NOS) leads to the total abolition of the inhibitory effect of EPO. While nerve fibers and innervating endothelium express NOS (Wang et al., 1995), these results strongly suggest that the glomus cells are the source of EPO-mediated NO production in our preparations. Moreover, these results are in line with previous findings suggesting that NO is produced by glomus cells mitochondria (Gozal et al., 1996; Dvorakova and Kummer, 2005; Li et al., 2010; Atanasova et al., 2016), and works as competitor inhibitor of the mitochondrial complex IV (Cooper and Brown, 1995; Cleeter et al., 1994; Castello, 2006; Basu, 2008). Furthermore, considering that NO is a neuronal inhibitory messenger in the carotid body (Wang et al., 1995; Fung et al., 2001; Moya et al., 2012), it has been suggested that a mitochondrial dysfunction inducing overproduction of NO in the glomus cells of CB may lead to hypoventilation in permanent residents of high altitude, which in the long run could result in chronic mountain sickness (CMS) (Holmes, 2018). It would also be worth investigating whether high concentrations of EPO could induce the formation of S-nitrosothiols that pack into vesicles and are released in response to hypoxia or hypercapnia (Lipton et al., 2001). On the other hand, it should be noted that the effect of EPO on CSN activity does not depend solely on NO production, EPO also promotes dopamine production (Yamamoto et al., 2000), therefore EPO-related CSN activation could be mediated by dopamine.

Our results, although derived from *ex-vivo* data, allow us to conceptualize the importance of EPO dose to the CSN response. The concentration of EPO in the blood of residents (animals and humans) at sea level is very small, of the order of 0.006 IU/ml (Jelkmann, 2007), and of healthy high-altitude residents, 0.023 IU/ml (Basu et al., 2007). Exposure to hypoxia (3,500–4,000 m above sea level) does not raise EPO levels higher than 0.035 IU/ml in lowlanders. Even the highest levels of blood EPO reported in patients with chronic mountain sickness are around 0.042 IU/ml (Villafuerte et al., 2014). Only in this last group, we could think that circulating EPO is related to the inhibited response of the carotid body in these patients. However, high levels of EPO are administered in cases of anemia (around 300–500 IU/kg) (Santoro and Canova, 2005), doses of between 60 and 350 IU/kg are administered for doping purposes (Jelkmann and Lundby, 2011), and the highest doses of EPO are used to promote neuroprotection in stroke cases, for example, with levels between 10 k and 40 k IU/kg (Mcpherson and Juul, 2008). Thus, the results obtained in this work are very relevant for clinics and suggest that the administration of high EPO concentrations could supress chemoreceptor response to hypoxia. On the other hand, the physiological role of EPO and NO in CB remains elusive. However, since the erythropoietic and respiratory neural control systems are inversely activated (increased erythropoiesis, decreased ventilation, and vice versa), it is tempting to suggest that EPO and NO in the CB act as essential factors for connecting and balancing these systems. Thus, our results also suggest that EPO and NO protect the respiratory neural control system against physiological (such as at high altitude) and/or pathological (such as sleep apnea and hypertension) hyperactivation.

In conclusion, EPO has a dose-dependent modulation of CSN activity under conditions of hypoxia and hypercapnia. Concentrations below 0.5 IU/ml of EPO (concentrations observed in physiological conditions) stimulate CSN activity under hypoxia, whereas concentrations above 0.5 IU/ml (therapeutic and doping concentrations) could inhibit CSN activity under both hypoxic and hypercapnic stimuli by apparent excessive secretion of NO. Thus, application of very high doses of EPO suggests caution, especially when patients are exposed to hypoxic conditions. Despite the physiological role of EPO and NO in CB remains elusive, our results suggest that these factors would protect the respiratory control system from hyperactivation occurring in condition of physiological (such as at high altitude) and/or pathological hypoxia.

## Data Availability

The raw data supporting the conclusions of this article will be made available by the authors, without undue reservation.
